# First record of two Leptothecata medusae (Cnidaria, Hydrozoa) in Colombia with annotations on their distribution and ecology

**DOI:** 10.3897/BDJ.13.e138523

**Published:** 2025-01-20

**Authors:** Jorge L Llorente-Vega, Cristina Cedeño-Posso, Jorge A. Quirós-Rodríguez

**Affiliations:** 1 University of Córdoba, Faculty of Basic Sciences, Department of Biology, Biotechnology Research Group (GRUBIODEC), Montería, Colombia University of Córdoba, Faculty of Basic Sciences, Department of Biology, Biotechnology Research Group (GRUBIODEC) Montería Colombia; 2 Marine and Coastal Research Institute (INVEMAR), Biodiversity and Marine Ecosystems Program, Inventory, Taxonomy and Biology of Species, Santa Marta, Colombia Marine and Coastal Research Institute (INVEMAR), Biodiversity and Marine Ecosystems Program, Inventory, Taxonomy and Biology of Species Santa Marta Colombia; 3 University of Córdoba, Faculty of Basic Sciences, Department of Biology, Natural Products Chemistry Research Group (PRONAT), Montería, Colombia University of Córdoba, Faculty of Basic Sciences, Department of Biology, Natural Products Chemistry Research Group (PRONAT) Montería Colombia

**Keywords:** biodiversity, ecology, estuary, invasive species, zooplankton

## Abstract

**Background:**

Hydromedusae are a group of planktonic cnidarians that represent the medusoid phase in the life cycle of most members of the class Hydrozoa, whose primary function is to produce and release gametes. These organisms are generally small and translucent, with slight pigmentation, except for those that inhabit great depths and exhibit the typical body shape of a jellyfish. In Colombia, studies on this group are limited due to the scarcity of updated taxonomic information and the small number of expert scientists. The most recent inventory records 71 species in the waters of the Pacific and Atlantic Oceans.

**New information:**

In this study, we report for the first time the presence of *Eucheilotaduodecimalis* and *Eutoninascintillans* in Colombia, based on 28 zooplankton samples collected from seven stations between February and May 2024, using horizontal tows with a simple conical zooplankton net. Our research emphasises the significance of scientific exploration in new geographic regions and provides valuable data on the distribution and ecology of these species, contributing to a deeper understanding of their population dynamics.

## Introduction

Hydromedusae represent the pelagic adult stage in the life cycle of most hydrozoans. Their primary function is to produce and release gametes which, after fertilisation, give rise to the planula larva. This larva can develop into a sexual medusa, an asexual polyp (solitary or colonial) or intermediate stages resembling “actinuloid” larvae (Bouillon et al. 2006, Goffredo and Dubinsky 2016). It is worth noting that some hydrozoan species lack the medusa stage, while others, such as those in the orders Narcomedusae and Trachymedusae, lack the polyp stage (Bouillon 1999, Mendoza-Becerril et al. 2019).

From an ecological perspective, this group of organisms occupy one of the top levels of the food web as predators, influencing the structure and population dynamics of various zooplankton groups (Andrade 2019a, Ahuatzin-Hernández et al. 2020, Tosetto et al. 2023). Additionally, certain species are considered indicators of ocean current dynamics, as their limited swimming capacity makes them susceptible to being carried by water flows to areas where their presence is unusual (Ramírez and Zamponi 1981, Baldrich and López 2014).

Despite this, studies and records on hydromedusae in Colombia are scarce. For the Pacific, the works of Baldrich and López (2010), Baldrich and López (2014) and López and Baldrich (2014) are available, while for the Colombian Caribbean, studies by Medellín-Mora and Martínez-Ramírez (2010), Cedeño-Posso (2014) and Dueñas et al. (2019) have been conducted. Additionally, the most recently published inventory provides a taxonomic list of 71 recorded species (Oliveira et al. 2016). Therefore, in this paper, we document for the first time in Colombia the presence of *Eucheilotaduodecimalis* (A. Agassiz, 1862) and *Eutoninascintillans* (Bigelow, 1909).

Our findings underscore the importance of continuing scientific exploration in regions lacking records of planktonic hydrozoans, as well as in areas with heavy maritime traffic, such as ports and shipping routes. These zones are key entry points for invasive species, often introduced through ballast water discharge and hull fouling (Apín-Campos and Torres-Pérez 2016, Abelando 2021). Research in these areas is crucial for monitoring biodiversity changes, understanding species dispersal and developing strategies to mitigate the ecological impacts of invasive species, thereby helping to protect native ecosystems (Gracia et al. 2011, Arregocés and Cañón 2015).

## Materials and methods

**Figure 1.**
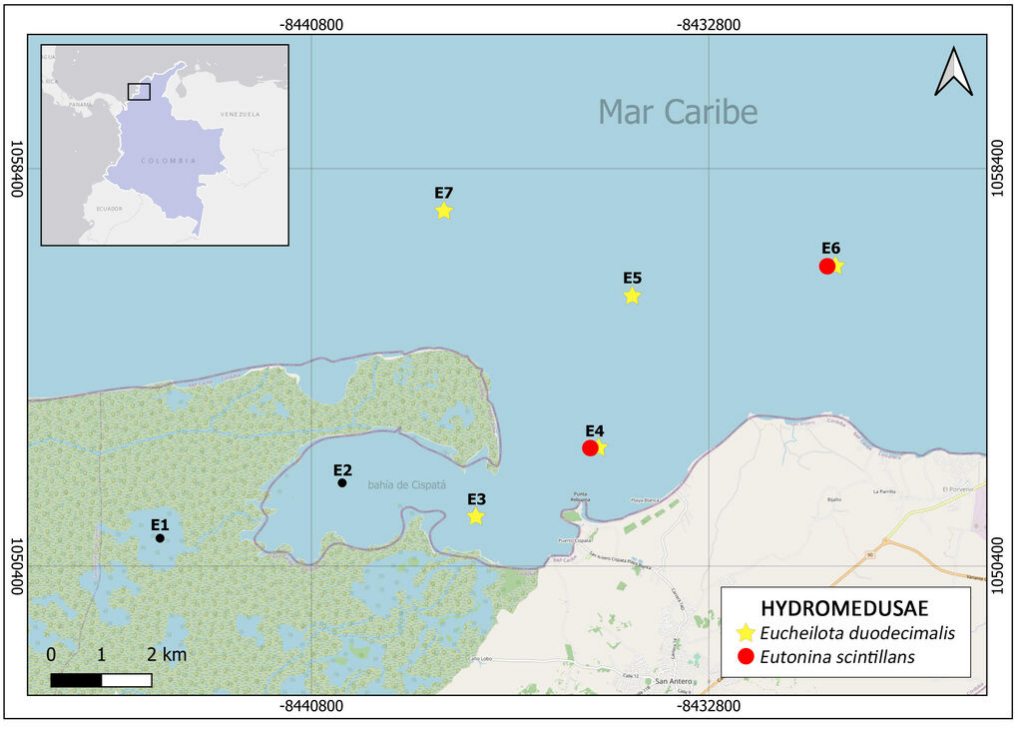
Study area and location of sampling stations.

Material was collected during four sampling campaigns conducted in February and May 2024 at seven sampling stations in the coastal area of San Antero, Colombian Caribbean (Fig. 1). A total of 28 samples were obtained through semicircular tows at depths of 0 to 3 metres, at a speed of 1.5 knots for five minutes. A simple conical zooplankton net with a 250 μm mesh size, 150 cm length, 40 cm mouth diameter and a 250 ml collecting container was used for sample collection.

Samples were narcotised in situ with 10% magnesium chloride, fixed in 5% formalin and neutralised with 1 g·l⁻¹ sodium tetraborate (Cedeño-Posso et al. 2024). Hydromedusae were sorted using a Luxeo 6Z stereomicroscope with a built-in digital camera and a Zefir Cpad optical microscope with an LCD screen. Subsequently, they were identified following the taxonomic keys provided by Bouillon (1999), Bouillon et al. (2006), Rodríguez et al. (2007) and Wang et al. (2018).

The examined specimens were deposited in the Marine Invertebrates Section of the Zoology Laboratory Collection at the University of Córdoba (LZUC-CD) as reference material. The catalogue numbers for each lot are provided in the materials examined section. Finally, information on the distribution and ecology of the species in question was compiled from published articles and books. Additionally, given the scarcity of studies, records from Masters' and Doctoral theses were included as potential observations of the species; however, these should be interpreted with caution.

## Taxon treatments

### Eucheilota duodecimalis

(A. Agassiz, 1862)

F3C0C73B-B82F-541E-B245-C79C6CEF7964

*Phialiumdodecasema* (Haecke, 1879).
*Phialiumduodecimalis* (A. Agassiz, 1862).

#### Materials

- **Type status:** Other material. **Occurrence:** individualCount: 2; occurrenceID: F2E14B0B-E093-5DAE-8314-DF7D10B71423; **Taxon:** kingdom: Animalia; phylum: Cnidaria; class: Hydrozoa; order: Leptothecata; family: Lovenellidae; genus: Eucheilota; **Location:** country: Colombia; stateProvince: Córdoba; municipality: San Antero; locality: E3; verbatimDepth: 0.5-3 m; verbatimCoordinates: 9°24'11.47"N, 75°47'43.63"W; **Event:** eventDate: 02-04-2024; **Record Level:** collectionCode: LZUC-CD. C-0105
- **Type status:** Other material. **Occurrence:** individualCount: 93; occurrenceID: A6F45DF1-82B6-5ECF-A360-8308142F2CAE; **Location:** country: Colombia; stateProvince: Córdoba; municipality: San Antero; locality: E4; verbatimDepth: 0.5-3 m; verbatimCoordinates: 9°24'55.02"N, 75°46'25.32"W; **Event:** eventDate: 02-26-2024/05-19-2024
- **Type status:** Other material. **Occurrence:** individualCount: 178; occurrenceID: A17D4637-EC8E-518F-9456-2172D947F833; **Location:** country: Colombia; stateProvince: Córdoba; municipality: San antero; locality: E5; verbatimDepth: 05-3 m; verbatimCoordinates: 9°26'33.73"N, 75°46'1.20"W; **Event:** eventDate: 02-26-2024/05-19-2024
- **Type status:** Other material. **Occurrence:** individualCount: 120; occurrenceID: E9B9905F-DAAF-523B-BA67-4B22BE70207F; **Location:** country: Colombia; stateProvince: Córdoba; municipality: San Antero; locality: E6; verbatimDepth: 0.5-3 m; verbatimCoordinates: 9°26'52.79"N, 75°43'51.47"W; **Event:** eventDate: 02-26-2024/05-19-2024
- **Type status:** Other material. **Occurrence:** individualCount: 121; occurrenceID: 0CCF5262-5FD5-5EBE-AD64-C86AE6F6983E; **Location:** country: Colombia; stateProvince: Córdoba; municipality: San antero; locality: E7; verbatimDepth: 0.5-3 m; verbatimCoordinates: 9°27'28.14"N, 75°48'4.10"W; **Event:** eventDate: 02-26-2024/05-19-2024

#### Description

**Figure 2.**
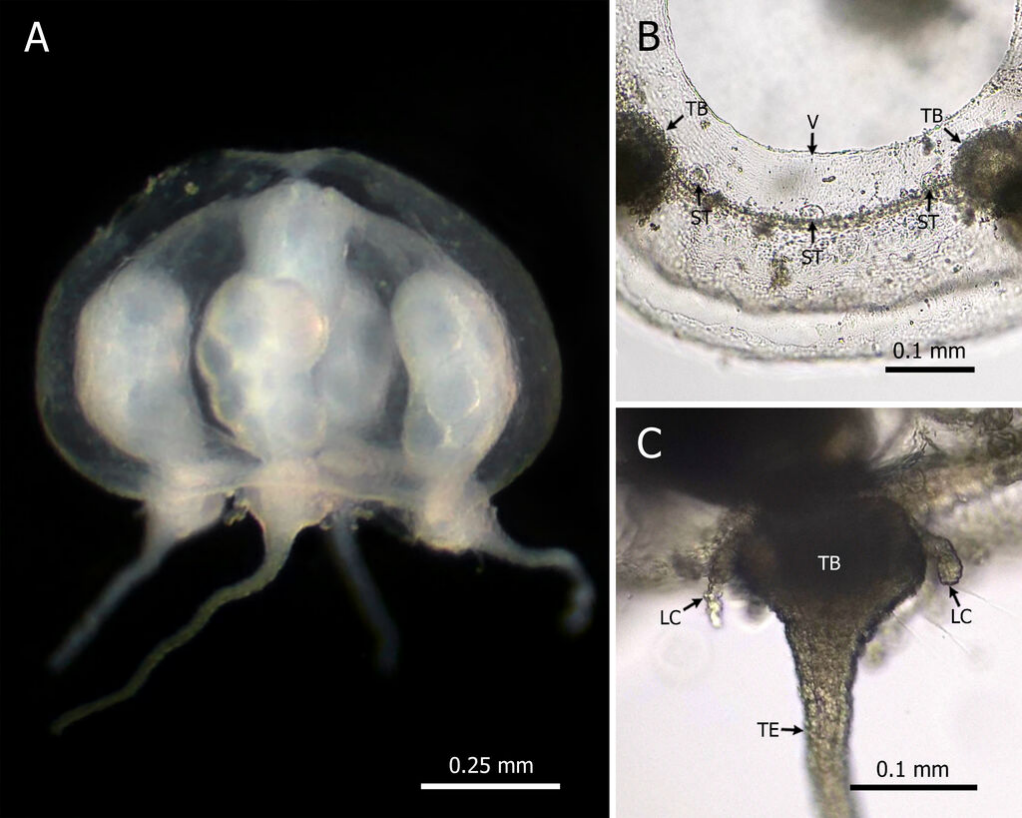
*Eucheilotaduodecimalis* (A. Agassiz, 1862). **(A)** Lateral view; **(B)** Detail of a quadrant of the umbrelar margin; **(C)** Tentacular bulb. Abbreviations: LC = lateral cirri; ST = statocyst; TB = tentacular bulb; TE = tentacle; V = velum.

Umbrella hemispherical, 0.3-1.1 mm in height and 0.1-1.5 mm in width; mesoglea uniform; manubrium short and narrow; four simple radial canals; four interradial “gonads” located in the distal region of the radial canals, near the tentacular swellings. Mature small medusae were recorded with developed germ sacs; broad velum; four large, conical tentacular bulbs, each with a hollow tentacle and one to three pairs of lateral cirri; 12 closed statocysts, three per quadrant, two flanking the tentacular bulbs and one positioned inter-radially (Fig. 2).

#### Diagnosis

Four tentacles, with conical tentacular bulbs with a pair of lateral cirri; 12 statocysts, three per quadrant; marginal warts absent (Agassiz 1862, Bouillon 1999, Wang et al. 2018).

#### Distribution

**Figure 3.**
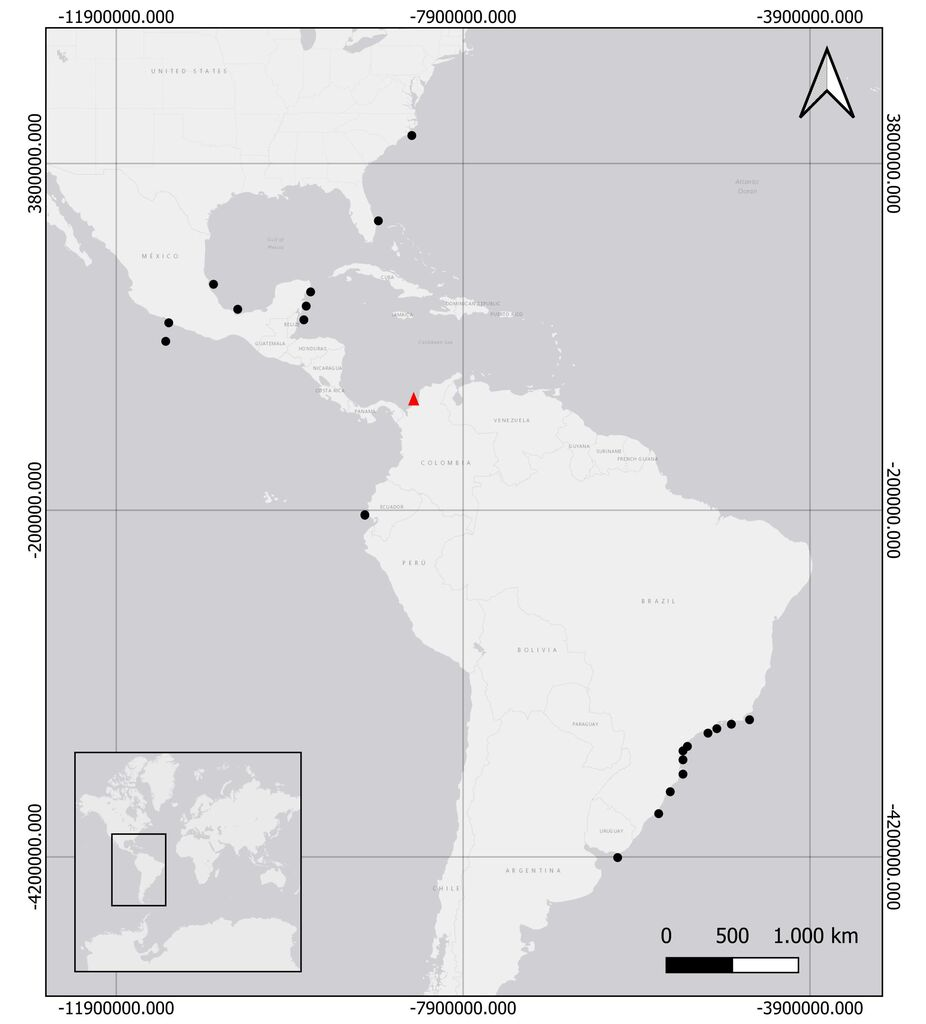
Distribution of *Eucheilotaduodecimalis* (A. Agassiz, 1862). black circles = previous records; red triangle = new locality in Colombia.

Western Atlantic, from the United States to southern Mexico and South America, from Brazil to Uruguay (Tronolone 2001, Migotto et al. 2002, Tronolone 2007, Nagata et al. 2014, Oliveira et al. 2016 and Ríos 2018). Pacific Ocean, in Ecuador and Mexico (Segura-Puertas et al. 2003, Mujica and Andrade 2019) (See Fig. 3).

#### Taxon discussion

The observed specimens have a mean size of 0.6 mm in height and 1.0 mm in diameter, which are smaller than sizes reported by other authors (2.5 mm in diameter), such as Kramp (1961) and Bouillon (1999), but similar to that documented by Nagata et al. (2014) (1.3 mm in diameter). In general, *E.duodecimalis* shows very low morphological variability. The analysed specimens were consistent with the original descriptions provided by Agassiz (1862), Bouillon (1999) and Wang et al. 2018.

### Eutonina scintillans

(Bigelow, 1909)

A45AB9E5-4792-5936-8AF5-D27D568AA33A

*Eutimalphesscintillans* (Bigelow, 1909).

#### Materials

- **Type status:** Other material. **Occurrence:** individualCount: 2; occurrenceID: 8F1FF918-C383-5C5C-A489-AE0902C265B6; **Taxon:** kingdom: Animalia; phylum: Cnidaria; class: Hydrozoa; order: Leptothecata; family: Eirenidae; genus: Eutonina; **Location:** country: Colombia; stateProvince: Córdoba; municipality: San antero; locality: E4; verbatimDepth: 0.5-3 m; verbatimCoordinates: 9°24'55.02"N, 75°46'25.32"W; **Event:** eventDate: 19-05-2024; **Record Level:** collectionCode: LZUC-CD. C-0104
- **Type status:** Other material. **Occurrence:** individualCount: 1; occurrenceID: A32585A0-8ED0-5FF5-9160-FF22245A95B2; **Location:** country: Colombia; stateProvince: Córdoba; municipality: San antero; locality: E6; verbatimDepth: 0.5-3 m; verbatimCoordinates: 9°26'52.79"N, 75°43'51.47"W; **Event:** eventDate: 02-04-2024

#### Description

**Figure 4.**
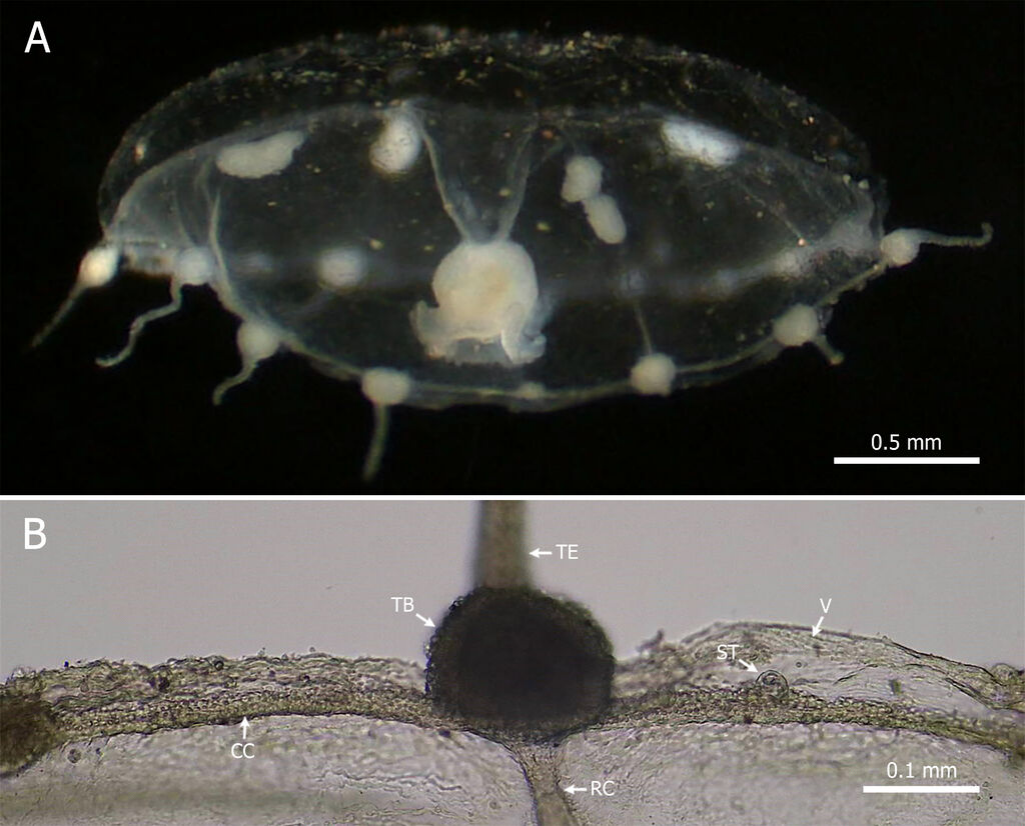
*Eutoninascintillans* (Bigelow, 1909). **(A)** Lateral view; **(B)** Detail of the umbrella margin. Abbreviations: CC = circular canal; RC = radial canal; ST = statocyst; TB = tentacular bulb; TE = tentacle; V = velum.

Umbrella 1.1-2.7 mm in height and 1.1-2.3 mm in width; mesoglea thick; peduncle short and manubrium globose; mouth with four crenulated lips; four subumbrellar gonads occupying one-fourth of the radial canals, separate from the peduncle; velum narrow; 16 marginal tentacles with conical basal bulbs; eight closed statocysts, two per quadrant; no cirri or marginal warts (Fig. 4).

#### Diagnosis

Short peduncle; globular manubrium; mouth with simple or crenulated lips; four subumbrellar gonads located in the distal portion of the radial canals; 12 to 30 marginal tentacles; eight closed statocysts; no cirri or marginal warts (Bigelow 1909, Goy 1979, Bouillon 1999, Bouillon et al. 2004, Rodríguez et al. 2007).

#### Distribution

**Figure 5.**
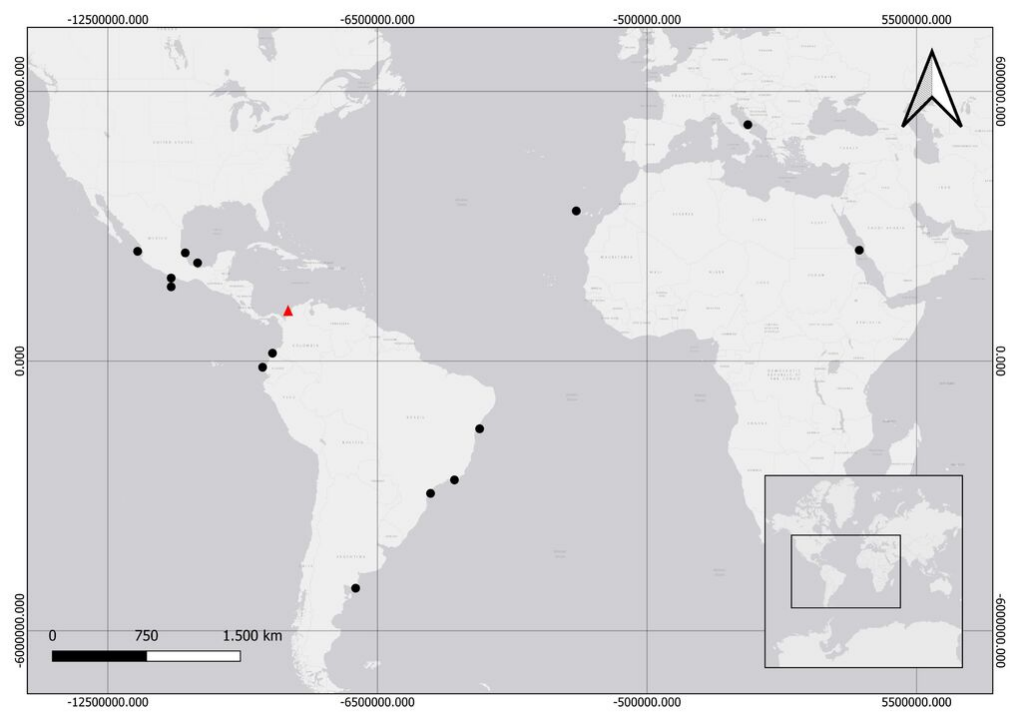
Distribution of *Eutoninascintillans* (Bigelow, 1909). black circles = previous records; red triangle = new locality in Colombia.

Atlantic Ocean (Goy 1979, Migotto et al. 2002, Rodríguez et al. 2007, Genzano et al. 2008, Oliveira et al. 2016, Ríos 2018); Pacific Ocean, in Mexico and Ecuador (Kramp 1961, Segura-Puertas et al. 2003, Andrade 2019b, Andrade 2021); Mediterranean, Adriatic and Red Seas, as well as on the east coast of Africa (Kramp 1968, Bouillon et al. 2004, Thibault-Botha et al. 2013) (See Fig. 5).

#### Taxon discussion

The average size of the analysed specimens (1.3 mm in height and 2.8 mm in diameter) is smaller than that reported by other authors (5–10 mm in height and width), such as Kramp (1961), Bouillon (1999) and Rodríguez et al. (2007), but similar to that documented by Goy (1979). Additionally, each specimen contains 16 marginal tentacles, consistent with the findings of Rodríguez et al. (2007). Based on the high degree of morphological plasticity of the species, Goy (1979) identified three common characteristics present in all descriptions of *E.scintillans*, all of which were observed in the specimens examined.

## Discussion

Based on the most recent review of hydromedusa species in Colombia (Oliveira et al. 2016), the family Lovenellidae is represented by *Eucheilotacomata* (Bigelow, 1909) and *E.menoni* (A. Agassiz, 1862) and the family Eirenidae by *Eirenelactea* (Mayer, 1900), *E.viridula* (Péron & Lesueur, 1809), *Eutimagentiana* (Haeckel, 1879), *E.mira* (McCrady, 1859) and *Phialopsisdiegensis* (Torrey, 1909). Therefore, the two species documented in this study represent new records for the waters of Colombia.

*Eucheilotaduodecimalis* is a common species in coastal areas influenced by inland waters. It has been documented in the United States, Mexico, Brazil, Uruguay and Ecuador (Tronolone 2001, Migotto et al. 2002, Segura-Puertas et al. 2003, Tronolone 2007, Nagata et al. 2014, Oliveira et al. 2016, Ríos 2018 and Andrade 2019a). Therefore, this study represents the first report of this species in Colombia (Fig. 3).

The available ecological information on *E.duodecimalis* indicates that it is an exclusively neritic species with euryhaline behaviour (Vannucci 1963). In our study, its presence was observed at five sampling stations between February and May, within a salinity range of 28 to 35 PSU. These findings are consistent with those documented by Bardi (2011), Nogueira et al. (2018) and Nascimento et al. (2019), who reported the species in salinities of 15-30 in south-eastern and southern Brazil, 17.8-31.5 in south-eastern Brazil and 15-34 PSU in Paranaguá (Brazil), respectively. Similarly, for the Pacific Region, Mujica and Andrade (2019) reported specimens at salinities of 32.3 to 35.8 PSU in Santa Elena (Ecuador), while Ríos (2018) documented them in ranges of 27-31.92 and 27-29.85 PSU in Veracruz, Mexico.

These studies have generally reported the collection of numerous specimens (more than 30), except for Bardi (2011), who obtained a total of eight specimens. Additionally, the absence of a homogeneous distribution pattern has been noted, suggesting that the local dispersal of the species may be influenced by a combination of biotic and abiotic factors specific to each area. Furthermore, Bardi (2011), Nogueira et al. (2018) and Nascimento et al. (2019) agree that *E.duodecimalis* exhibits a low seasonal trend. However, in this study, a significant increase in the number of specimens collected was observed in May. Therefore, we suggest that a more extensive collection of data is required to establish more precisely the relationship between its abundance and distribution and the prevailing climatic conditions.

*Eutoninascintillans* exhibits a broad and widely dispersed distribution. In the Atlantic Ocean, it has been reported along the coasts of Brazil, Argentina and México (Goy 1979, Migotto et al. 2002, Rodríguez et al. 2007, Genzano et al. 2008, Oliveira et al. 2016, Ríos 2018). In the Pacific Ocean, it has been recorded in Mexico and Ecuador (Kramp 1961, Segura-Puertas et al. 2003, Andrade 2019b, Andrade 2021). Furthermore, its presence has been documented in the Mediterranean, Adriatic and Red Sea, as well as along the eastern coast of Africa (Kramp 1968, Bouillon et al. 2004, Thibault-Botha et al. 2013). Here, we record for the first time its presence in Colombia and the Caribbean Sea (Fig. 5).

According to Rodríguez et al. (2007), Oliveira et al. (2016) and Ríos (2018), *E.scintillans* is a neritic species that inhabits estuarine ecosystems, with its local distribution directly influenced by salinity. The specimens analysed in this study were captured at two sampling stations during April and May, in waters with salinity levels of 30 and 33 PSU. These findings are consistent with those reported by Rodríguez et al. (2007), Ríos (2018) and Andrade (2021), who documented the species in areas with salinity levels of 33.2 PSU in Costa Bonaerense (Buenos Aires, Argentina), 24-29.85 PSU in Veracruz (Mexico) and 33 PSU in La Libertad and Manta (Ecuador), respectively. However, the variations observed amongst the studies may be attributed to the influence of multiple factors and the unique dynamics of each ecosystem.

Likewise, Rodríguez et al. (2007) proposed that *E.scintillans* may be an introduced species in certain regions, likely facilitated by maritime vectors such as ballast water discharge or other anthropogenic nautical activities. This hypothesis is supported by the appearance of new isolated populations. However, comprehensive ecological and genetic studies are needed to clarify the mechanisms driving this expansion, assess the potential impacts on native communities and gain a better understanding of the species' population dynamics, including its reproductive strategies, dispersal mechanisms and habitat preferences in these new environments.

## Supplementary Material

XML Treatment for Eucheilota duodecimalis

XML Treatment for Eutonina scintillans
